# Genome sequence of the *Trifolium rueppellianum* -nodulating *Rhizobium leguminosarum* bv. *trifolii* strain WSM2012.

**DOI:** 10.4056/sigs.4528262

**Published:** 2013-12-15

**Authors:** Wayne Reeve, Vanessa Melino, Julie Ardley, Rui Tian, Sofie De Meyer, Jason Terpolilli, Ravi Tiwari, Ronald Yates, Graham O’Hara, John Howieson, Mohamed Ninawi, Brittany Held, David Bruce, Chris Detter, Roxanne Tapia, Cliff Han, Chia-Lin Wei, Marcel Huntemann, James Han, I-Min Chen, Konstantinos Mavromatis, Victor Markowitz, Ernest Szeto, Natalia Ivanova, Natalia Mikhailova, Ioanna Pagani, Amrita Pati, Lynne Goodwin, Tanja Woyke, Nikos Kyrpides

**Affiliations:** 1 Centre for Rhizobium Studies, Murdoch University, Western Australia, Australia; 2 Department of Agriculture and Food, Western Australia, Australia; 3 DOE Joint Genome Institute, Walnut Creek, California, USA; 4 Los Alamos National Laboratory, Bioscience Division, Los Alamos, New Mexico, USA; 5 Biological Data Management and Technology Center, Lawrence Berkeley National Laboratory, Berkeley, California, USA

**Keywords:** root-nodule bacteria, nitrogen fixation, rhizobia, *Alphaproteobacteria*

## Abstract

*Rhizobium leguminosarum* bv. *trifolii* WSM2012 (syn. MAR1468) is an aerobic, motile, Gram-negative, non-spore-forming rod that was isolated from an ineffective root nodule recovered from the roots of the annual clover *Trifolium rueppellianum* Fresen growing in Ethiopia. WSM2012 has a narrow, specialized host range for N_2_-fixation. Here we describe the features of *R. leguminosarum* bv. *trifolii* strain WSM2012, together with genome sequence information and annotation. The 7,180,565 bp high-quality-draft genome is arranged into 6 scaffolds of 68 contigs, contains 7,080 protein-coding genes and 86 RNA-only encoding genes, and is one of 20 rhizobial genomes sequenced as part of the DOE Joint Genome Institute 2010 Community Sequencing Program.

## Introduction

Atmospheric dinitrogen (N_2_) is fixed by specialized soil bacteria (root nodule bacteria or rhizobia) that form non-obligatory symbiotic relationships with legumes. The complex, highly-evolved legume symbioses involve the formation of specialized root structures (nodules) as a consequence of a tightly controlled mutual gene regulated infection process that results in substantial morphological changes in both the legume host root and infecting rhizobia [1]. When housed within root nodules, fully effective N_2_-fixing bacteroids (the N_2_-fixing form of rhizobia) can provide 100% of the nitrogen (N) requirements of the legume host by symbiotic N_2_-fixation.

Currently, N_2_-fixation provides ~40 million tonnes of nitrogen (N) annually to support global food production from ~300 million hectares of crop, forage and pasture legumes in symbioses with rhizobia [2]. The most widely cultivated of the pasture legumes is the legume genus *Trifolium* (clovers). This genus inhabits three distinct centers of biodiversity with approximately 28% of species in the Americas, 57% in Eurasia and 15% in Sub-Saharan Africa [3]. A smaller subset of about 30 species, almost all of Eurasian origin, are widely grown as annual and perennial species in pasture systems in Mediterranean and temperate regions [3]. Globally important commonly cultivated perennial species include *T. repens* (white clover*), T. pratense* (red clover*), T. fragiferum* (strawberry clover) and *T. hybridum* (alsike clover). *Trifolium rueppellianum* is an important annual self-pollinating species grown in the central African continent as a food and forage legume.

Clovers usually form N_2_-fixing symbiosis with the common soil bacterium *Rhizobium leguminosarum* bv. *trifolii*, and different combinations of *Trifolium* spp. hosts and strains of *R. leguminosarum* bv. *trifolii* can vary markedly in symbiotic compatibility [4] resulting in a broad range of symbiotic development outcomes ranging from ineffective (non-nitrogen fixing) nodulation to fully effective N_2_-fixing partnerships [5].

*Rhizobium leguminosarum* bv. *trifolii* strain WSM2012 (syn. MAR1468) has a narrow, specialized host range for N_2_ fixation [6] and was isolated from a nodule recovered from the roots of the annual clover *T. rueppellianum* growing in Ethiopia in 1963. This strain is a good representative of one of the six centers of biodiversity, Africa, and can be used to investigate the evolution and biodiversity of *R. leguminosarum* bv. *trifolii* strains [6]. Here we present a preliminary description of the general features for *R. leguminosarum* bv. *trifolii* strain WSM2012 together with its genome sequence and annotation.

## Classification and general features

**Figure 1:**
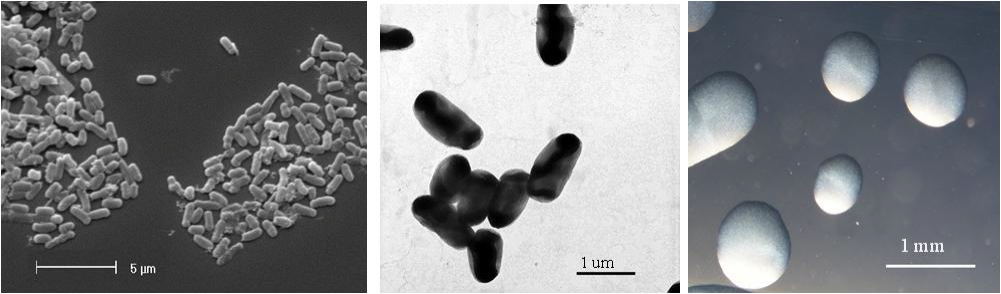
Images of *Rhizobium leguminosarum* bv. *trifolii* strain WSM2012 using scanning (Left) and transmission (Center) electron microscopy as well as light microscopy to visualize the colony morphology on a solid medium (Right).

**Figure 2:**
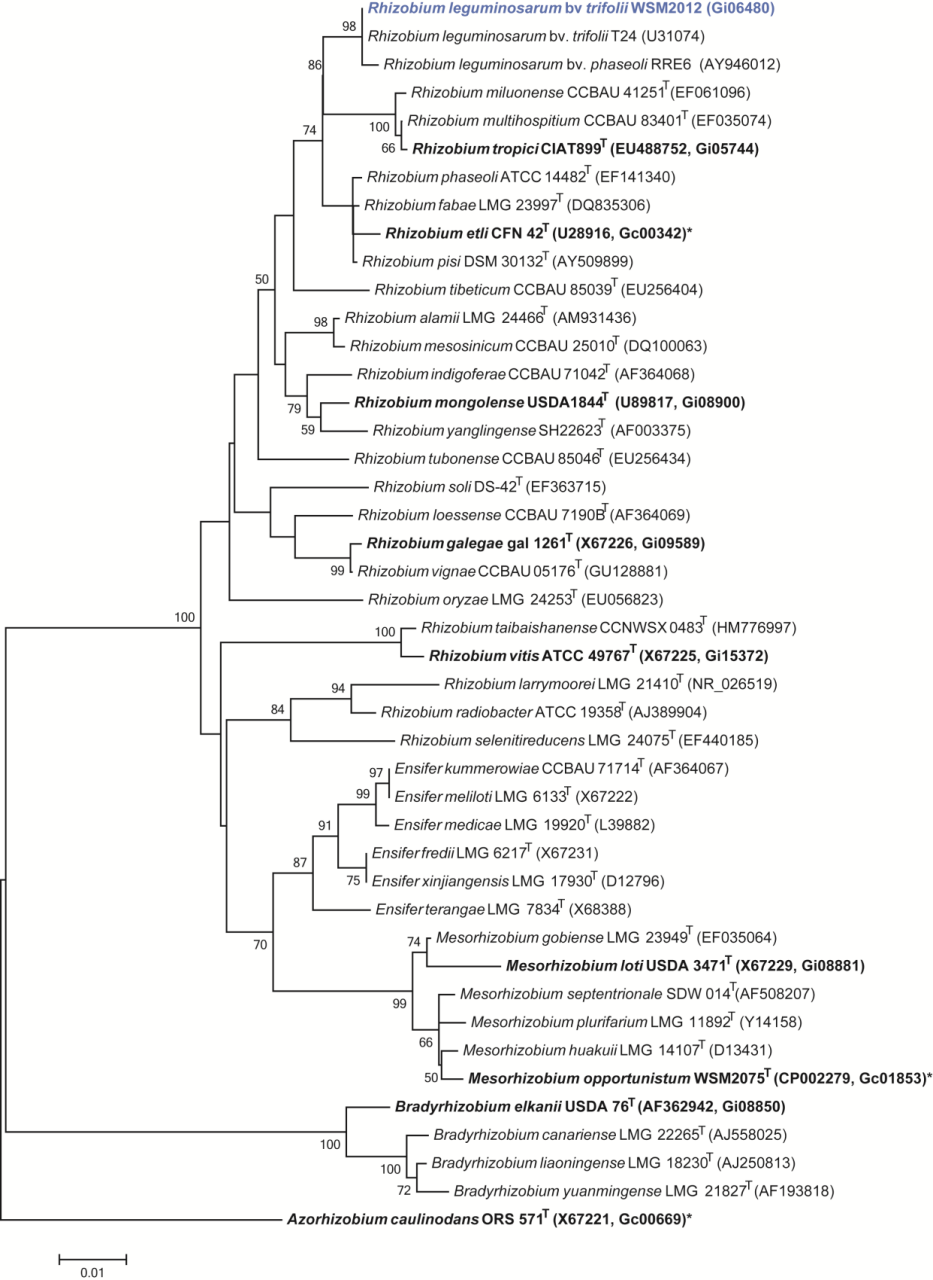
Phylogenetic tree showing the relationship of *Rhizobium leguminosarum* bv. *trifolii* WSM2012 (shown in blue print) with some of the root nodule bacteria in the order *Rhizobiales* based on aligned sequences of the 16S rRNA gene (1,306 bp internal region). All sites were informative and there were no gap-containing sites. Phylogenetic analyses were performed using MEGA, version 5.05 [23]. The tree was built using the maximum likelihood method with the General Time Reversible model. Bootstrap analysis [24] with 500 replicates was performed to assess the support of the clusters. Type strains are indicated with a superscript T. Strains with a genome sequencing project registered in GOLD [25] are in bold print and the GOLD ID is mentioned after the accession number. Published genomes are indicated with an asterisk.

*R. leguminosarum* bv. *trifolii* strain WSM2012 is a motile, Gram-negative rod (Figure 1 Left and Center) in the order *Rhizobiales* of the class *Alphaproteobacteria*. It is fast growing, forming colonies within 3-4 days when grown on half Lupin Agar (½LA) [7] at 28°C. Colonies on ½LA are white-opaque, slightly domed, moderately mucoid with smooth margins (Figure 1 Right). Minimum Information about the Genome Sequence (MIGS) is provided in Table 1. Figure 2 shows the phylogenetic neighborhood of *R. leguminosarum* bv. *trifolii* strain WSM2012 in a 16S rRNA sequence based tree. This strain clusters closest to *Rhizobium leguminosarum* bv. *trifolii* T24 and *Rhizobium leguminosarum* bv. *phaseoli* RRE6 with 99.9% and 99.8% sequence identity, respectively.

**Table 1:** Classification and general features of *Rhizobium leguminosarum* bv. *trifolii* WSM2012 according to the MIGS recommendations [8]

**MIGS ID**	**Property**	**Term**	**Evidence code**
	Current classification	Domain *Bacteria*	TAS [9]
		Phylum *Proteobacteria*	TAS [10]
		Class *Alphaproteobacteria*	TAS [11,12]
		Order *Rhizobiales*	TAS [12,13]
		Family *Rhizobiaceae*	TAS [14,15]
		Genus *Rhizobium*	TAS [14,16-19]
		Species *Rhizobium leguminosarum* bv. *trifolii*	TAS [14,16,19,20]
	
	Gram stain	Negative	IDA
	Cell shape	Rod	IDA
	Motility	Motile	IDA
	Sporulation	Non-sporulating	NAS
	Temperature range	Mesophile	NAS
	Optimum temperature	28°C	NAS
	Salinity	Non-halophile	NAS
MIGS-22	Oxygen requirement	Aerobic	NAS
	Carbon source	Varied	IDA
	Energy source	Chemoorganotroph	NAS
MIGS-6	Habitat	Soil, root nodule, on host	IDA
MIGS-15	Biotic relationship	Free living, symbiotic	IDA
MIGS-14	Pathogenicity	Non-pathogenic	NAS
	Biosafety level	1	TAS [21]
	Isolation	Root nodule	IDA
MIGS-4	Geographic location	Ethiopia	IDA
MIGS-5	Nodule collection date	April 1963	IDA
MIGS-4.1 MIGS-4.2	Longitude Latitude	40.209961 9.215982	IDA
MIGS-4.3	Depth	Not recorded	
MIGS-4.4	Altitude	Not recorded	

Evidence codes – IDA: Inferred from Direct Assay; TAS: Traceable Author Statement (i.e., a direct report exists in the literature); NAS: Non-traceable Author Statement (i.e., not directly observed for the living, isolated sample, but based on a generally accepted property for the species, or anecdotal evidence). These evidence codes are from the Gene Ontology project [22].

### Symbiotaxonomy

*R. leguminosarum* bv. *trifolii* WSM2012 nodulates (Nod^+^) and fixes N_2_ effectively (Fix^+^) with both the African annual clover *T. mattirolianum* Chiov. and the African perennial clovers *T. cryptopodium* Steud. ex A. Rich and *T. usamburense* Taub [6]. WSM2012 is Nod^+^ Fix^-^ with the Mediterranean annual clover *T. subterraneum* L. and *T. glanduliferum* Boiss. and with both the African perennial clover *T. africanum* Ser. and the African annual clovers *T. decorum* Chiov. and *T. steudneii* Schweinf [1,26]. WSM2012 does not nodulate (Nod^-^) with the Mediterranean annual clover *T. glanduliferum* Prima nor the South American perennial clover *T. polymorphum* Poir [6].

## Genome sequencing and annotation information

### Genome project history

This organism was selected for sequencing on the basis of its environmental and agricultural relevance to issues in global carbon cycling, alternative energy production, and biogeochemical importance, and is part of the Community Sequencing Program at the U.S. Department of Energy, Joint Genome Institute (JGI) for projects of relevance to agency missions. The genome project is deposited in the Genomes OnLine Database [25] and an improved-high-quality-draft genome sequence in IMG. Sequencing, finishing and annotation were performed by the JGI. A summary of the project information is shown in Table 2.

**Table 2:** Genome sequencing project information for *Rhizobium leguminosarum* bv. *trifolii* strain WSM2012.

**MIGS ID**	**Property**	**Term**
MIGS-31	Finishing quality	Improved high-quality draft
MIGS-28	Libraries used	Illumina GAii shotgun and paired end 454 libraries
MIGS-29	Sequencing platforms	Illumina, 454 GS FLX Titanium technologies
MIGS-31.2	Sequencing coverage	7.4× 454 paired end, 300× Illumina
MIGS-30	Assemblers	Velvet 1.013, Newbler 2.3, phrap 4.24
MIGS-32	Gene calling methods	Prodigal 1.4, GenePRIMP
	GOLD ID	Gi06480
	NCBI project ID	65301
	Database: IMG	2509276033
	Project relevance	Symbiotic N_2_ fixation, agriculture

### Growth conditions and DNA isolation

*Rhizobium leguminosarum* bv. *trifolii* strain WSM2012 was grown to mid logarithmic phase in TY rich medium [27] on a gyratory shaker at 28°C. DNA was isolated from 60 ml of cells using a CTAB (Cetyl trimethyl ammonium bromide) bacterial genomic DNA isolation method [28].

### Genome sequencing and assembly

The genome of *Rhizobium leguminosarum* bv. *trifolii* strain WSM2012 was sequenced at the Joint Genome Institute (JGI) using a combination of Illumina [29] and 454 technologies [30]. An Illumina GAii shotgun library which produced 63,969,346 reads totaling 4,861.7 Mb, and a paired end 454 library with an average insert size of 8 Kb which produced 428,541 reads totaling 92.6 Mb of 454 data were generated for this genome. All general aspects of library construction and sequencing performed at the JGI can be found at the JGI user homepage [28]. The initial draft assembly contained 158 contigs in 6 scaffolds. The 454 paired end data was assembled with Newbler, version 2.3. The Newbler consensus sequences were computationally shredded into 2 Kb overlapping fake reads (shreds). Illumina sequencing data were assembled with Velvet, version 1.0.13 [31], and the consensus sequences were computationally shredded into 1.5 Kb overlapping fake reads (shreds). The 454 Newbler consensus shreds, the Illumina VELVET consensus shreds and the read pairs in the 454 paired end library were integrated using parallel phrap, version SPS - 4.24 (High Performance Software, LLC). The software Consed [32-34] was used in the following finishing process. Illumina data were used to correct potential base errors and increase consensus quality using the software Polisher developed at JGI (Alla Lapidus, unpublished). Possible mis-assemblies were corrected using gapResolution (Cliff Han, unpublished), Dupfinisher [35], or sequencing cloned bridging PCR fragments with subcloning. Gaps between contigs were closed by editing in Consed, by PCR and by Bubble PCR (J-F Cheng, unpublished) primer walks. A total of 167 additional reactions were necessary to close gaps and to raise the quality of the finished sequence. The estimated genome size is 6.7 Mb and the final assembly is based on 49.8 Mb of 454 draft data which provides an average 7.4× coverage of the genome and 2,010 Mb of Illumina draft data which provides an average 300× coverage of the genome.

### Genome annotation

Genes were identified using Prodigal [36] as part of the DOE-JGI Annotation pipeline [37], followed by a round of manual curation using the JGI GenePRIMP pipeline [38]. The predicted CDSs were translated and used to search the National Center for Biotechnology Information (NCBI) non-redundant database, UniProt, TIGRFam, Pfam, PRIAM, KEGG, COG, and InterPro databases. These data sources were combined to assert a product description for each predicted protein. Non-coding genes and miscellaneous features were predicted using tRNAscan-SE [39], RNAMMer [40], Rfam [41], TMHMM [42], and SignalP [43]. Additional gene prediction analyses and functional annotation were performed within the Integrated Microbial Genomes (IMG-ER) platform [44].

## Genome properties

**Figure 3:**
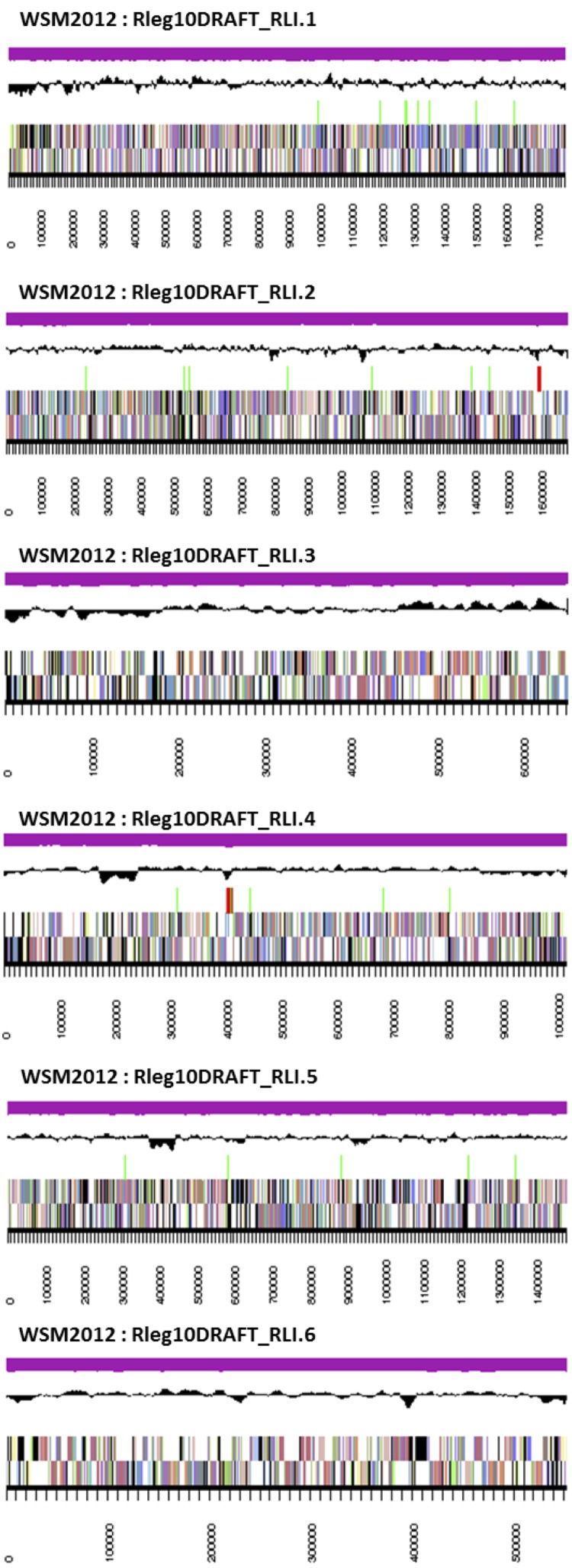
Graphical map of the genome of *Rhizobium leguminosarum* bv. *trifolii* strain WSM2012. From bottom to the top of each scaffold: Genes on forward strand (color by COG categories as denoted by the IMG platform), Genes on reverse strand (color by COG categories), RNA genes (tRNAs green, sRNAs red, other RNAs black), GC content, GC skew.

The genome is 7,180,565 nucleotides with 60.89% GC content (Table 3) and comprised of 6 scaffolds (Figure 3) of 68 contigs. From a total of 7,166 genes, 7,080 were protein encoding and 86 RNA only encoding genes. The majority of genes (72.87%) were assigned a putative function while the remaining genes were annotated as hypothetical. The distribution of genes into COGs functional categories is presented in Table 4.

**Table 3:** Genome Statistics for *Rhizobium leguminosarum* bv. *trifolii* WSM2012

**Attribute**	**Value**	**% of Total**
Genome size (bp)	7,180,565	100.00
DNA coding region (bp)	6,196,449	86.29
DNA G+C content (bp)	4,372,528	60.89
Number of scaffolds	6	
Number of contigs	68	
Total gene	7,166	100.00
RNA genes	86	1.20
rRNA operons*	3	
Protein-coding genes	7,080	98.80
Genes with function prediction	5,222	72.87
Genes assigned to COGs	5,682	79.29
Genes assigned Pfam domains	5,892	82.22
Genes with signal peptides	615	8.58
Genes with transmembrane helices	1,617	22.56
CRISPR repeats	0	

*1 extra 5s rRNA gene

**Table 4:** Number of protein coding genes of *Rhizobium leguminosarum* bv. *trifolii* WSM2012 associated with the general COG functional categories.

**Code**	**Value**	**%age**	**COG Category**
J	206	3.25	Translation, ribosomal structure and biogenesis
A	0	0.00	RNA processing and modification
K	619	9.76	Transcription
L	237	3.74	Replication, recombination and repair
B	2	0.03	Chromatin structure and dynamics
D	48	0.76	Cell cycle control, mitosis and meiosis
Y	0	0.00	Nuclear structure
V	77	1.21	Defense mechanisms
T	330	5.20	Signal transduction mechanisms
M	335	5.28	Cell wall/membrane biogenesis
N	85	1.34	Cell motility
Z	1	0.02	Cytoskeleton
W	0	0.00	Extracellular structures
U	108	1.70	Intracellular trafficking, secretion and vesicular transport
O	187	2.95	Posttranslational modification, protein turnover, chaperones
C	327	5.16	Energy production conversion
G	636	10.03	Carbohydrate transport and metabolism
E	716	11.29	Amino acid transport metabolism
F	107	1.69	Nucleotide transport and metabolism
H	215	3.39	Coenzyme transport and metabolism
I	214	3.37	Lipid transport and metabolism
P	311	4.90	Inorganic ion transport and metabolism
Q	154	2.43	Secondary metabolite biosynthesis, transport and catabolism
R	802	12.65	General function prediction only
S	625	9.85	Function unknown
-	1,484	20.71	Not in COGS
